# Longitudinal association between housing accessibility and activities of daily living: the role of self-efficacy and control in people ageing with Parkinson’s disease

**DOI:** 10.1186/s12877-020-01574-z

**Published:** 2020-05-25

**Authors:** Giedre Gefenaite, Jonas Björk, Susanne Iwarsson, Björn Slaug, Steven M. Schmidt, Maria H. Nilsson

**Affiliations:** 1 Department of Health Sciences, Faculty of Medicine, Lund University, Box 157, 221 00 Lund, Sweden; 2 Clinical Studies Sweden, Forum South, Skåne University Hospital, Lund, Sweden; 3 Division of Occupational and Environmental Medicine, Lund University, Lund, Sweden; 4 Clinical Memory Research Unit, Department of Clinical Sciences Malmö, Lund University, Lund, Sweden; 5 Memory Clinic, Skåne University Hospital, Malmö, Sweden

**Keywords:** ADL, General self-efficacy, Housing-related control beliefs, Housing accessibility

## Abstract

**Background:**

External housing-related control beliefs (HCB) and general self-efficacy (GSE) influence different health outcomes in the general ageing population, but there is no information of their role in people ageing with Parkinson’s disease (PD). This study aimed to longitudinally assess the role of external HCB and GSE on the association between housing accessibility and activities of daily living (ADL) among people ageing with PD.

**Methods:**

Baseline and 3-year follow-up data on 130 community-living participants from the Swedish project ‘Home and Health in People Ageing with PD’ were collected. Assessments addressed housing accessibility, external HCB, GSE, generic ADL and ADL specific to PD. The moderating effects of external HCB and GSE were assessed by including an interaction term in multivariable logistic regression.

**Results:**

There were statistically significant interactions between housing accessibility and GSE on ADL (*p* = 0.03), and housing accessibility and external HCB on PD specific ADL (*p* = 0.03). After stratifying the analyses by GSE, housing accessibility problems led to more dependence and difficulty in ADL in participants with low GSE (OR 1.14; 95% CI 1.02–1.28). After stratifying by external HCB, housing accessibility increased dependence and difficulty in PD specific ADL in participants with low external HCB (OR 1.35; 95% CI 1.03–1.76).

**Discussion:**

The results suggest that housing accessibility predicts ADL in people with PD with GSE and external HCB playing a moderating role for generic ADL and ADL specific to PD, respectively. Further longitudinal studies should validate these findings and explore their potential application in PD-related care and rehabilitation.

## Background

Parkinson’s disease (PD) is the second most common neurodegenerative disease [1]. Among people 60 years and older, its prevalence is estimated at about 1%, with an age-related increase [2]. People living and ageing with PD experience increasing challenges in daily life due to the progressive nature of the disease. In addition to the challenging motor symptoms (e.g. bradykinesia, rigidity, tremor, and postural instability), non-motor symptoms (e.g. sleep disturbances and autonomous dysfunction) also add to the disease burden [3]. PD is associated with a death of dopamine-producing cells, but other neurotransmitter systems are also affected (e.g. serotonin) [4]. Already when newly diagnosed, people with PD show limitations in activities of daily living (ADL) and decreased quality of life [5]. A prior study suggested that people with self-reported PD have more housing accessibility related problems and perceive their homes as less usable than matched controls without PD due to more functional limitations [6].

Housing accessibility is an indicator of person-environment fit [7, 8] and can be defined as an interaction between the person’s functional capacity (P) and the demands of the physical environment (E) [9]. Housing accessibility problems have been associated with ADL dependence in people 67–84 years old in general [10], but evidence for this linkage is lacking in specific population groups such as people with PD.

A previous study showed that people with PD with more functional limitations and difficulties in ADL tended to rely more on external control (i.e., influences of others, luck, chance or faith, etc.) in order to manage their housing situation [11]. General self-efficacy (GSE; i.e., beliefs in one’s abilities to achieve a goal) [12] was shown to play a role in health-related outcomes; it affected physical activity and self-management in people with PD [13, 14]. More specifically, lower self-efficacy was found to be a barrier for exercising [15], while higher general and balance self-efficacy were associated with more life satisfaction [16] and better walking capacity [17]. There is some evidence that assigning control over one’s housing plays a moderating role in the association between housing accessibility problems and ADL in adults 80–89 years old [18], but not in those 67–70 years old [19], which suggests age related differences. Previous studies were cross-sectional and conducted in the general population limiting causal inference and generalizability to vulnerable populations, such as those ageing with specific chronic diseases. As yet, there are no longitudinal studies that measured the role of assigning control over one’s housing or GSE in the housing context.

Given the existing evidence about housing accessibility, its effects on ADL dependence, with potentially moderating effects of control assigned over one’s housing and GSE, exploring these associations further in the PD population could provide useful insights for improving and facilitating treatment, care, rehabilitation, housing adaptations and housing provision. The aim of this study was to longitudinally assess the role of external HCB and GSE on the association between housing accessibility and ADL among people ageing with PD.

## Methods

### Study design and sample

We used data from the longitudinal ‘Home and Health in people ageing with PD’ (HHPD) cohort [20](T1; *N* = 255), which included baseline assessment and a 3-year follow-up (T2; *N* = 164). The participants (ICD-10 diagnosis G20.9 for at least one year) were recruited from departments specialized in PD from three hospitals in the Skåne region, Sweden [21]. Difficulties in understanding or speaking Swedish, living outside Skåne County, severe cognitive difficulties or other reasons (e.g. hallucinations, recent stroke) that made a potential participant unable to give informed consent or take part in the majority of the data collection were applied as exclusion criteria [21]. In the current study, we excluded those without functional limitations at T1 (*n* = 10) or with missing data on ADL dependence and difficulty (*n* = 23) at T2 resulting in *N* = 131. In addition, three persons had missing scores on external HCB, resulting in *N* = 128 for the analysis testing the role of external HCB. One person had missing scores on GSE, resulting in a sample size of *N* = 130 to test the role of GSE.

### Primary outcome: generic ADL at T2

To capture generic ADL, the ADL Staircase [22] was used to assess five personal (bathing, dressing, going to the toilet, transfer, feeding) and four instrumental (transportation, cleaning, shopping, cooking) activities by interview and observation at the home visit. ADL items at T2 were assessed as independent, partly dependent or dependent, with dependence defined as assistance from another person. For those who were rated as independent in an activity, the data collector asked whether the participant performed the specific task with difficulty (yes/no) [23]. For each item, an ordinal variable based on the ADL Staircase assessment and perceived difficulty in ADL was created as independent with no difficulty (scored 0); independent with difficulty (scored 1); partly dependent (scored 2); or dependent (scored 3). The ADL Staircase is reliable and valid for the assessment of functional ability in people of 74–84 years old [10] as well as the general population [24]. To our knowledge, its psychometric properties have not been evaluated in people with PD. Because the assumptions to use the outcomes as continuous measures in linear regression models were not met for this study, generic ADL scores were dichotomised with a cut-off value of <=9, representing the lowest 33% of the scores at T2.

### Secondary outcome: ADL specific to PD at T2

ADL specific to PD was assessed at T2 with the self-reported Parkinson’s Diseases Activities of Daily Living Scale (PADLS) [25]. The respondents were asked to tick the one of five descriptions, which best described how Parkinson’s disease had affected their day-to-day activities in the last month (scores 1–5, higher = more difficulty/dependence). PADLS is a reliable and valid measure of ADL in people with PD [25, 26]. For this study, PADLS scores were dichotomised into no/mild difficulties (original scores 0–1 = score 0) or moderate/severe/extreme difficulties (original scores 3–5 = score 1), which is a principle used in previous studies [26, 27].

### Primary exposure: housing accessibility at T1

Housing accessibility at T1 was assessed with the Housing Enabler (HE) [28], an internationally acknowledged research instrument to assess housing accessibility, which has been used for over 20 years. A trained rater administered the personal (P) component assessing functional limitations (12 items) and dependence on mobility devices (two items) as present/not present by a combination of interview and observation. The environmental (E) component was assessed based on observations of the actual environment in a detailed rating of 161 environmental barriers as present/not present. Using instrument-specific software, a P × E interaction score for each individual was calculated, with E representing the barriers that generate severe/impossible accessibility problems [29]. Then, to separate the P × E interaction effect from the main effects of P and E, a novel measure of environmental barriers net of functional capacity was calculated as the residuals from linear regression with the natural logarithm of P as the independent variable and the interaction term of P × E as the dependent variable (for more detail see [29]). P × E residuals is a measure of how many more (or fewer) actual barriers than expected (given the number of functional limitations and the P × E scores they generate) each person had in their housing environment. This measure is called the relative accessibility problem score (RAPS) and is adjusted for P (i.e. functional limitations). RAPS was used as the main exposure at T1. To assess the risk for confounding, the RAPS was categorized into fewer barriers than expected, expected number of barriers and more barriers than expected. As cut-off for this categorization, a difference of at least four barriers more than or less than expected was chosen, as it has been shown in several other samples to give a fairly even distribution between the categories [29].

### Moderators: external HCB and GSE at T1

External HCB (i.e., beliefs that control over one’s housing is up to others, luck, chance or fate) at T1 were assessed by the self-reported Housing-related Control Beliefs Questionnaire’s (HCQ) scales of external control-powerful others (8 items) and external control-chance (8 items) [30]. Each statement is rated on a five-point scale (1 = I do not at all agree to 5 = I agree very much), higher scores indicating more perceived external control over one’s housing. In previous studies, HCQ showed comparable satisfactory internal consistency of the subscales in 66–69 and 65–91 year olds, and good test-retest reliability in 65–91 year olds in Germany [30]; internal consistency in the HHPD baseline sample was acceptable [31]. For the current study, the total score combining two scales was dichotomised at 50% cut-off into low [16–33] and high (34–80) external HCB.

GSE was assessed by the self-administered 10-item General self-efficacy Scale [32] at T1. Each item is rated on a four-point scale (not at all true = 1 to exactly true = 4). The GSE Scale has demonstrated satisfactory internal consistency, test-retest reliability and construct validity in people with PD in Sweden [33]. For the current study, the total score was dichotomised at a 50% cut-off into low [10–25] and high [26–40] GSE.

### Other covariates

Based on previous literature [26, 34–42], several socio-demographic and clinical characteristics were included in the analyses as covariates because they have shown associations with ADL and/or different aspects of housing in people with PD or older adults.

Socio-demographic characteristics were either collected from the medical records or self-reported, and included age (in years), sex (male/female), marital status (married/cohabitant; unmarried/divorced/widowed), education (university/college, lower), country where they lived most of their lives (Sweden/other), satisfaction with one’s financial situation (scale 0–10, higher score = more satisfaction), type of housing tenure (owning/renting) measured at T1, and relocation and housing adaptations (occurred/not occurred) between T1 and T2. Clinical characteristics included self-reported depressive symptoms assessed with the Geriatric Depression Scale (15 dichotomous items, scores 0–15, higher = more symptoms) [43] and PD duration (years). PD severity in relation to the off condition was based on a standardized interview according to Hoehn and Yahr (HY) (stages 1–5, higher = more difficulty) [44] at T1.

### Statistical analysis

The strength of correlations was assessed by Pearson’s and Spearman’s correlation coefficients (r_s_). The correlation coefficients were defined as very weak (0.0–0.2), weak (0.2–0.4), moderate (0.4–0.6), strong (0.6–0.8) and very strong (0.8–1). Differences across RAPS categories were tested by chi-square test, one-way analysis of variance (ANOVA) and median test for k samples.

The effects of the main exposure at T1 on the outcomes at T2 were assessed with multiple logistic regression. Further adjustment for any of the covariates associated with RAPS scores (Table 1) only changed the effect estimates marginally (with 10% as tolerance level [45]). The results of these further adjustments are therefore not presented.

**Table 1:** Descriptive characteristics according to the categorized RAPS (*N* = 130)

	Total	Fewer barriers than expected	Expected barriers	More barriers than expected	***p***-value
	n = 130 (100%)	*n* = 38 (29,2%)	*n* = 63 (48,5%)	*n* = 29 (22,3%)	
	**n (%)**	**n (%)**	**n (%)**	**n (%)**	**Chi-square test**
Sex (male), T1	84 (64.6)	27 (71.1)	43 (68.3)	14 (48.3)	0.11
Civil status (married/cohabiting), T1	85 (65.4)	27 (71.1)	44 (69.8)	14 (48.3)	0.09
Education (university/college), T1	47 (36.2)	18 (47.4)	23 (36.5)	6 (20.7)	0.08
Lived in Sweden for most of the time, T1	127 (97.7)	36 (94.7)	62 (98.4)	29 (100)	0.32
Own their home, T1	99 (76.2)	31 (81.6)	49 (77.8)	19 (65.5)	0.28
Moved since T1, T2	20 (15.4)	5 (13.2)	10 (15.9)	5 (17.2)	0.89
Housing adjustments since T1, T2	26 (20.6)	12 (32.4)	7 (11.1)	7 (26.9)	0.03
	**Mean (SD)**	**Mean (SD)**	**Mean (SD)**	**Mean (SD)**	**ANOVA**
Age, T1	68.3 (8.6)	70.1 (9.1)	66 (7.8)	71 (8.6)	0.01
External HCB, T1	38.8 (10.6)	39.4 (9.2)	36 (8.8)	44.2 (13.6)	0.003
GSE, T1	29.6 (6.1)	29.4 (5.8)	30.1 (6.4)	28.9 (6.1)	0.67
	**Median (Q1-Q3)**	**Median (Q1-Q3)**	**Median (Q1-Q3)**	**Median (Q1-Q3)**	**Median test (k samples)**
Satisfied with income, T1	7 (5–9)	7.5 (5.8–10)	7 (5–9)	7.5 (5–8)	0.97
PD duration, T1	8 (5–13)	8 (5–11.5)	7 (4–12.3)	10 (6.3–14.8)	0.64
PD severity, HY: off condition, T1	3 (2–4)	2 (1.8–4)	2 (2–3)	3.5 (3–4)	0.03
Depressive symptoms, T1	5 (5–7)	6 (5–7)	5 (5–6)	6 (5–7)	0.07

Note. *RAPS* relative accessibility problem score; *HCB* housing-related control beliefs; *GSE* general self-efficacy; *PD* Parkinson’s disease; *HY* Hoehn and Yahr scale; *ANOVA* one-way analysis of variance

To test the moderating role of external HCB and GSE, we regressed generic ADL or ADL specific to PD on RAPS, functional limitations, external HCB or GSE, and an interaction term with RAPS and external HCB or GSE, respectively. We calculated odds ratios (OR) and their 95% confidence intervals (CI). When the *p*-value for an interaction term was ≤0.05, the associations of the main models including RAPS adjusted for functional limitations on the outcomes were tested in different strata of the moderators.

The analysis was conducted in IBM SPSS Statistics 24.0 (Armonk, NY: IBM Corp).

### Comparison of the HHPD baseline sample to the follow-up

At T1, based on *N* = 130 sample (Table 1), 65% were men, 65% were married/cohabiting, 36% had university/college education, 98% lived in Sweden for most of their lives, were on average 68 years old (range 45–91) and 76% owned their dwelling. The median of depressive symptoms was 5 (i.e. according to GDS-15), PD duration was 8 years and median of PD severity in self-reported off condition according to the Hoehn and Yahr scale (HY) [44] was 3. Eighty-five percent did not move and 77% did not have housing adaptations between T1 and T2. With regard to these characteristics, our sample was nearly identical to the complete sample at T2 [46]. For more details on the recruitment, baseline and 3-year follow-up characteristics, see [20, 46].

## Results

Higher RAPS at T1 had very weak positive correlations with generic ADL (*p*-value > 0.05) and ADL specific to PD (*p*-value ≤0.05) at T2 (Table 2). Functional limitations at T1 were moderately positively correlated with more dependence in both generic ADL and ADL specific to PD at T2, and had weak positive and negative correlations with external HCB and GSE at T1 (*p*-value ≤0.01), respectively (Table 2). Both outcomes were moderately positively correlated (p-value ≤0.01), and external HCB was weakly negatively correlated with GSE (p-value ≤0.05) (Table 2).

**Table 2:** Correlations between the exposure, outcomes and potential moderators (*N* = 130)

	RAPS, T1	Functional limitations, T1	External HCB, T1	GSE, T1	Generic ADL, T2	ADL specific to PD, T2
RAPS, T1	1.00	0.09	0.15	−0.06	0.10	0.2*
Functional limitations, T1		1.00	0.42**	−0.24**	0.58**	0.54**
External HCB, T1			1.00	−0.22*	0.21*	0.19*
GSE, T1				1.00	−0.24**	−0.25**
Generic ADL, T2					1.00	0.67**
ADL specific to PD, T2						1.00

Note. *RAPS* relative accessibility problem score; *HCB* housing-related control beliefs; *GSE* general self-efficacy; *ADL* activities of daily living; *PD* Parkinson’s Disease. Pearson correlation coefficients are presented between continuous variables (RAPS, functional limitations, external HCB and GSE), while correlations with categorical generic ADL and ADL specific to PD are Spearman correlation coefficients

* *p*-value ≤0.05 level (2-tailed); ** *p*-value ≤0.01 level (2-tailed)

There were statistically significant interactions between RAPS and GSE on generic ADL (p-value = 0.03), and between RAPS and external HCB on ADL specific to PD (*p*-value = 0.03). As compared to models with RAPS and functional limitations only, when including the interaction terms between RAPS and GSE and RAPS and external HCB, the explained variance increased from 35 to 41% and from 32 to 37%, respectively (Tables 3 and 4). After stratifying the analyses by GSE, RAPS was associated with generic ADL in participants with low GSE (OR 1.14; 95% CI 1.02–1.28; *p*-value = 0.02), while there was no effect in the high GSE group (OR 0.93; 95% CI 0.84–1.04; p-value = 0.21). When stratifying the analyses by external HCB, RAPS increased the odds of difficulty in ADL specific to PD in participants with low external HCB (OR 1.35; 95% CI 1.03–1.76; p-value = 0.03), while there was no association in the high scorers (OR 1.05; 95% CI .98–1.11; p-value = 0.18).

**Table 3:** The effect of RAPS on generic ADL (Models A1–3 *N* = 128; Models B1–3 *N* = 130)

	Model A1	Model A2	Model A3	Model B1	Model B2	Model B3
	OR (95% CI)	OR (95% CI)	OR (95% CI)	OR (95% CI)	OR (95% CI)	OR (95% CI)
RAPS, T1	1.02 (0.96–1.09)	1.02 (0.96–1.09)	1.32 (0.96–1.81)	1.02 (0.96–1.09)	1.03 (0.97–1.09)	1.76 (1.09–2.81)*
Functional limitations, T1	2.4 (1.75–3.28)***	2.29 (1.66–3.15)***	2.31 (1.68–3.2)***	2.41 (1.76–3.3)***	2.5 (1.77–3.53)***	2.9 (1.9–4.41)***
External HCB, T1		1.04 (0.99–1.09)	1.05 (0.99–1.11)			
RAPS*External HCB			0.99 (0.99–1.00)			
GSE, T1					0.89 (0.81–0.98)*	0.88 (0.8–0.97)**
RAPS*GSE						0.98 (0.97–1.00)*
R square	0.35	0.36	0.37	0.35	0.38	0.41

Note. *RAPS* relative accessibility problem score; *ADL* activities of daily living; *HCB* housing-related control beliefs; *GSE* general self-efficacy; *OR* odds ratio; *CI* confidence interval

* *p*-value ≤0.05 level (2-tailed); ** *p*-value ≤0.01 level (2-tailed); *** *p*-value ≤0.001 level (2-tailed)

**Table 4:** The effect of RAPS on ADL specific to PD (Models A1–3 *N* = 128; Models B1–3 *N* = 130)

	Model A1	Model A2	Model A3	Model B1	Model B2	Model B3
	OR (95% CI)	OR (95% CI)	OR (95% CI)	OR (95% CI)	OR (95% CI)	OR (95% CI)
RAPS, T1	1.07 (1.00–1.13)*	1.06 (1.00–1.13)*	1.50 (1.09–2.06)*	1.06 (1.00–1.13)*	1.07 (1.00–1.13)*	1.29 (0.9–1.84)
Functional limitations, T1	2.03 (1.56–2.65)***	1.91 (1.46–2.50)***	1.98 (1.49–2.63)***	2.05 (1.58–2.67)***	2.08 (1.57–2.75)***	2.14 (1.59–2.88)***
External HCB, T1		1.05 (1.00–1.10)*	1.35 (1.06–1.71)**			
RAPS*External HCB			0.99 (0.99–1.00)*			
GSE, T1					0.89 (0.81–0.96)**	1.09 (0.74–1.6)
RAPS*GSE						0.99 (0.98–1.01)
R square	0.32	0.34	0.37	0.32	0.36	0.37

Note**.***RAPS* relative accessibility problem score; *ADL* activities of daily living; *PD* Parkinson’s Disease; *HCB* housing-related control beliefs; *GSE* general self-efficacy; *OR* odds ratio; *CI* confidence interval

* *p*-value ≤0.05 level (2-tailed); ** *p*-value ≤0.01 level (2-tailed); *** *p*-value ≤0.001 level (2-tailed)

## Discussion

This longitudinal study showed that external HCB and GSE played a role when it comes to the influence of the housing accessibility on ADL in people ageing with PD. Specifically, higher RAPS was associated with a 13% increase in generic ADL scores among those with lower GSE, and a 35% increase in ADL specific to PD among the participants with lower external HCB. The present study is novel within the field as it explores longitudinal associations in home and health dynamics in people living and ageing with PD. While hitherto seldom reported in PD research, such knowledge is of importance as people are living and ageing with PD in their own homes for many years.

Two outcome measures for ADL were used in the current study, namely generic ADL measured by the ADL Staircase, and ADL specific to PD measured with PADLS. While the point estimates of RAPS on both outcomes when adjusted for functional limitations and the potential moderators were similar, only the association between RAPS and ADL specific to PD, and not the generic ADL, reached the predefined statistical significance (i.e., *p*-value ≤0.05). Moreover, the moderating effects of external HCB or GSE were not consistent across the outcomes. That is, the interaction between RAPS and external HCB reached the predefined statistical significance on ADL specific to PD, while the interaction between RAPS and GSE only appeared to be statistically significant for generic ADL. A potential explanation for these differences might be the operationalization of each of the outcomes. In ADL Staircase the level of dependence and difficulty for each of the nine ADL items is scored separately, and based on both interview and observation during the home visit [22]. It has been shown to meet validity and reliability requirements in the general population samples of older people [22, 23], but to our knowledge no studies have tested these psychometric properties in people with PD, who potentially also have other neurological conditions. PADLS is referred to as “a rough indicator of ADL” with satisfactory psychometric properties [26], and is based on self-report based on the past month where each score (i.e. 1–5) is described by rather complex examples referring to the level of dependence and difficulty. Its psychometric properties in people with PD, potentially with other underlying neurological conditions, to our knowledge is also absent. In addition to different structure of the instruments and the time frame of measurement, they do not address exactly the same ADL items in the same manner, and it is probably due to these differences that the correlation between the two instruments is moderate (r_s_ = 0.67). To summarize, the discrepancies probably reflect that these ADL assessments tap different ADL items and ways of assessment, and the participants might respond to different periods when being interviewed or having an explicit time frame to refer to.

The resulting moderation effects on housing and ADL dynamics might reflect that GSE and external HCB are different constructs that are not interchangeable. Self-efficacy is associated with locus of control [47], whereas general control beliefs are associated with external HCB [30]. A weak correlation (r_s_ = − 0.22) between external HCB and GSE was found in the present study, which indicates this difference. Furthermore, external HCB addresses proactivity specific to the home environment [30], while GSE is measuring one’s beliefs regarding coping with daily hassles in general [32]. One could speculate that because external HCB is part of perceived aspects of home [30], this construct might have a stronger association with perceived (i.e. self-reported) ADL (i.e. PADLS scores). Similarly, GSE and ADL Staircase are more universal and general measures, which is a potential explanation for finding that GSE had a moderating effect on generic ADL only. Differences of the moderating effects of external HCB and GSE and the use of different ADL measurements is therefore necessary to consider in future studies to be able to elucidate these discrepancies.

The results point to that GSE may be an important indicator of whether the housing environment affects dependence and difficulty in ADL. More specifically, the results suggest that we should be more proactive towards housing accessibility problems in people with PD who have low general self-efficacy as well as acknowledge self-efficacy in clinical PD-care and rehabilitation. Another study based on baseline HHPD data also showed that GSE was associated with perceived life satisfaction in people with PD [16], which underscores the importance of assessing its role in the housing context in this population subgroup.

In the Swedish younger old, no moderating effect of external HCB was found in relation to the ADL Staircase [19]. In very old people from Germany and Sweden those with a higher magnitude of accessibility problems and higher external HCB were more dependent in ADL according to ADL Staircase (*p*-value = 0.10) [18]. In the current study, RAPS increased ADL specific to PD only among those with low external HCB. Taken together these findings point to potentially different role of external HCB in housing and ADL dynamics across the studied population groups. However, the results cannot be directly compared due to different study designs (cross-sectional versus longitudinal). Also, in the above mentioned studies, housing accessibility was assessed by HE, while in the present study, additional steps were taken to separate the main effects of functional limitations and environmental barriers [29], resulting in RAPS. It would also be of interest to do similar studies in people with PD-dementia, atypical parkinsonism and dementia with Lewy bodies, as the role of external HCB and GSE in these groups might be different as well.

### Strengths

This is the first longitudinal study investigating the links between housing accessibility and health-related outcomes in people living and ageing with PD. Additionally, the role of GSE in the housing context and its effects on generic ADL as well as ADL specific to PD has never been tested before. In addition, while there were some missing data, our study sample was representative of the original follow-up HHPD cohort [46].

Until recently, the measure commonly used to capture housing accessibility problems with HE had a major analytical limitation. As the variance of HE is mostly attributed to the functional limitations or (personal) component, it hampered the possibility to estimate the effect of the environmental component on health outcomes [10]. Recently, a new analytic approach to separate the main effects of the personal and environmental components from their interaction effect was proposed, that is, using RAPS scores [29]. This approach enabled us to estimate the effect of environmental barriers on health outcomes, which provides a more adequate representation when addressing the housing environment and ADL.

### Limitations

One of the main limitations of this study is the small sample size. That is, we were not able to run analyses stratified by exposure and the moderators, which would have facilitated the interpretation of the results and more precise comparison to previous studies in the very old in the general population [18]. We were also not able to take into account the underlying comorbidities that are common in people with PD.

Due to the nature of the continuous RAPS score and that it could not be generated for those without functional limitations, we excluded 10 participants who had no functional limitations according to the HE, which limits the generalizability of the results. On the other hand, as the majority of people with PD do have functional limitations, overall the results are valid for the target population. Moreover, upon the presence of significant interactions, we stratified the sample for each of the moderators and were thus able to test the associations between RAPS and the outcomes in each stratum of interest.

Another limitation is that due to low internal consistency of the internal HCB [30], the latter was not available in HHPD, which puts a strain on the comprehensiveness of the housing-related control measure as such.

## Conclusions

The results suggest that housing accessibility predicts ADL in people with PD with GSE and external HCB playing a moderating role for generic ADL and ADL specific to PD, respectively. Further longitudinal studies should validate these findings and continue to explore these intriguing mechanisms and their potential application in PD-related care and rehabilitation.

## Data Availability

Anonymized data will be shared by request from a qualified academic investigator for the sole purpose of replicating procedures and results presented in the article and as long as data transfer is in agreement with EU legislation on the general data protection regulation and decisions by the Ethical Review Board of Sweden and Region Skåne.

## Abbreviations

ADL: Activities of daily living
GSE: General self-efficacy
HCB: Housing-related control beliefs
HHPD: Home and Health in people ageing with Parkinson’s disease
PD: Parkinson’s disease
RAPS: Relative accessibility problem score
